# Darcy-Forchheimer flow with Cattaneo-Christov heat flux and homogeneous-heterogeneous reactions

**DOI:** 10.1371/journal.pone.0174938

**Published:** 2017-04-05

**Authors:** Tasawar Hayat, Farwa Haider, Taseer Muhammad, Ahmed Alsaedi

**Affiliations:** 1 Department of Mathematics, Quaid-I-Azam University, Islamabad, Pakistan; 2 Nonlinear Analysis and Applied Mathematics (NAAM) Research Group, Department of Mathematics, Faculty of Science, King Abdulaziz University, Jeddah, Saudi Arabia; University of South Carolina, UNITED STATES

## Abstract

Here Darcy-Forchheimer flow of viscoelastic fluids has been analyzed in the presence of Cattaneo-Christov heat flux and homogeneous-heterogeneous reactions. Results for two viscoelastic fluids are obtained and compared. A linear stretching surface has been used to generate the flow. Flow in porous media is characterized by considering the Darcy-Forchheimer model. Modified version of Fourier's law through Cattaneo-Christov heat flux is employed. Equal diffusion coefficients are employed for both reactants and auto catalyst. Optimal homotopy scheme is employed for solutions development of nonlinear problems. Solutions expressions of velocity, temperature and concentration fields are provided. Skin friction coefficient and heat transfer rate are computed and analyzed. Here the temperature and thermal boundary layer thickness are lower for Cattaneo-Christov heat flux model in comparison to classical Fourier's law of heat conduction. Moreover, the homogeneous and heterogeneous reactions parameters have opposite behaviors for concentration field.

## 1. Introduction

Several industrial and environmental systems like geothermal energy systems, heat exchanger design, geophysics and catalytic reactors involve the convection flow subject to porous medium. The non-Darcian porous medium is the modified form of classical Darcian model which includes the inertia and boundary features. Flow subject to porous media is quite useful in building thermal insulation materials, beds of fossil fuels, energy storage units, nuclear waste disposal, solar receivers, heat exchanger, petroleum resources and numerous others [1–3]. The available literature witnesses that considerable attention has been given to modeling and analysis for flow subject to Darcy expression. The classical Darcy's law is valid under limited range of low velocity and smaller porosity. The Darcy's law is inadequate when inertial and boundary effects are accounted at higher flow rate. Due to such conditions it is impossible to ignore the effects of inertia and boundary. Forchheimer [4] included a square velocity term in the expression of Darcian velocity to predict the inertia and boundary features. Muskat [5] named this term as "Forchheimer term" which is always valid for high Reynolds number. Seddeek [6] examined the impacts of thermophoresis and viscous dissipation in Darcy-Forchheimer mixed convective flow saturating non-Darcy porous medium. Pal and Mondal [7] employed the Darcy-Forchheimer theory to examine the hydromagnetic flow of variable viscosity liquid in a porous medium. Sadiq and Hayat [8] analyzed the Darcy-Forchheimer flow of Maxwell liquid bounded by a convectively heated surface. Shehzad et al. [9] employed the Darcy-Forchheimer flow of variable thermal conductivity Oldroyd-B liquid past a vertical sheet with nonlinear convection and heat flux through Cattaneo-Christov theory. Recently Hayat et al. [10] studied the Darcy-Forchheimer flow of Maxwell fluid subject to variable thermal conductivity and heat flux through Cattaneo-Christov theory.

At present the researchers are engaged in analyzing the mechanism of heat transfer as a wave rather than diffusion due to its enormous applications in nanofluid mechanics and skin burns [11–18]. Heat transfer is a natural process which occurs due to difference of temperature between the system or different components of same system. The fundamental law of heat conduction suggested by Fourier [19] is frequently employed for heat transfer characteristics from the time it showed up in literature. But one of the major limitations of this model is that it leads to a parabolic energy equation which means that an initial disturbance would instantly experience by the system under consideration. This fact is referred in literature as “paradox of heat conduction”. To overcome this limitation, Cattaneo [20] modified this law by including relaxation time term. This term overcomes the paradox of heat conduction. Christov [21] further modified the Cattaneo theory [20] by changing the time derivative with Oldroyd upper-convected derivative. This theory is termed as Cattaneo-Christov heat flux theory. Straughan [22] used heat flux expression by Cattaneo-Christov theory to analyze the thermal convection in horizontal layer of viscous liquid. Ciarletta and Straughan [23] showed the structural stability and uniqueness of solutions for temperature equation by employing heat flux through Cattaneo-Christov theory. Haddad [24] studied thermal instability in Brinkman porous media by employing heat flux through Cattaneo-Christov expression. Mustafa [25] utilized the Cattaneo-Christov heat flux expression for rotating flow and heat transfer of Maxwell fluid. Hayat et al. [26] studied impact of Cattaneo-Christov heat flux in flow past a stretchable surface with variable thickness. Hayat et al. [27] also performed a comparative study for flows of viscoelastic materials by considering heat flux through Cattaneo-Christov expression. Waqas et al. [28] employed the heat flux by Cattaneo-Christov theory to examine the flow of variable thermal conductivity generalized Burgers fluid. Li et al. [29] studied the MHD viscoelastic flow and heat transfer past a vertical stretchable surface with Cattaneo-Christov heat flux. Recently Hayat et al. [30] analyzed three-dimensional flow of nanofluid subject to Cattaneo-Christov double diffusion.

Numerous chemically reacting structures involve the homogeneous and heterogenous reactions for example in catalysis, combustion and biochemical processes. Relation between the homogeneous and heterogeneous reactions is very complicated. There are various reactions which have the ability to progress slowly or not at all except in presence of a catalyst. Chemical reactions are employed in applications like fog formation and dispersion, food processing, ceramics and polymer production, hydrometallurgical industry and several others. Merkin [31] examined the homogeneous and heterogeneous reactions in flow of viscous liquid. He studied the homogeneous reaction for cubic autocatalysis and heterogeneous reaction on the catalyst surface. Homogenous and heterogeneous reactions with equal diffusivities are discussed by Chaudhary and Merkin [32]. Homogeneous-heterogeneous reactions in the stagnation-point flow towards a stretchable sheet are reported by Bachok et al. [33]. Homogeneous-heterogeneous reactions in flow of nanoliquid bounded by a porous stretchable surface is discussed by Kameswaran et al. [34]. Hayat et al. [35] studied melting heat in stretched flow of carbon nanotubes with homogeneous-heterogeneous reactions. Imtiaz et al. [36] explored unsteady magnetohydrodynamic flow due to a curved stretchable surface with homogeneous and heterogeneous reactions. Hayat et al. [37] reported Cattaneo-Christov heat flux effect in Jeffrey fluid flow subject to homogeneous-heterogeneous reactions. Hayat et al. [38] also examined the effects of homogeneous-heterogeneous reactions in flow of nanofluids over a nonlinear stretchable sheet having variable thickness. Sajid et al. [39] explored the homogeneous-heterogeneous reactions in magnetohydrodynamic nanofluid flow by a curved surface. Tanveer et al. [40] analyzed the homogeneous-heterogeneous reactions in mixed convective peristaltic flow of Sisko fluid. Recently Hayat et al. [41] studied the homogeneous-heterogeneous reactions and convective conditions in boundary layer flow of nanofluid by a stretching cylinder embedded in a porous medium.

Main objective of this investigation is to construct a mathematical model for Darcy-Forchheimer boundary layer flow of viscoelastic fluids past a linear stretching surface. Flow models for elastico-viscous and second grade fluids [42–48] are taken into account. Impacts of Cattaneo-Christov heat flux and homogeneous-heterogeneous reactions are also studied. Boundary layer approach is used in the mathematical development. Appropriate variables lead to strong nonlinear ordinary differential system. Convergent series solutions for velocity, temperature and concentration fields are developed by using optimal homotopy analysis method (OHAM) [49–58]. The contributions of various pertinent parameters are studied and discussed. Further the skin friction coefficient and local Nusselt number have been computed and analyzed through numerical data.

## 2. Modeling

Let us consider two-dimensional (2D) flow of viscoelastic fluids bounded by a linear stretching surface with constant surface temperature. Incompressible viscoelastic fluids saturate the porous space characterizing Darcy-Forchheimer model. Here *x* − axis is parallel to stretchable surface while *y* − axis normal to *x* − axis. Let *u*_*w*_(*x*) = *cx* describes the stretching velocity along the *x* − direction. Homogeneous and heterogeneous reactions of two chemical species *A* and *B* are accounted. Heat transfer mechanism is employed through Cattaneo-Christov heat flux theory. Homogeneous reaction for cubic autocatalysis is [36, 38]:

$$A+2B\to 3B,\;rate=k_{c}ab^{2},\;$$ (1)

while the heterogeneous reaction on the catalyst surface has been expressed by

$$A\to B,\;rate=k_{s}a,\;$$ (2)

where rate constants are denoted by *k*_*c*_ and *k*_*s*_ and the chemical species *A* and *B* have concentrations *a* and *b* respectively. The boundary layer equations governing the flow of viscoelastic fluids in the absence of viscous dissipation and thermal radiation can be written as follows [27, 38]:

$$\frac{\partial u}{\partial x}+\frac{\partial v}{\partial y}=0,\;$$ (3)

$$u\frac{\partial u}{\partial x}+v\frac{\partial u}{\partial y}=\nu \frac{\partial^{2}u}{\partial y^{2}}-k_{0}\left(u\frac{\partial^{3}u}{\partial x\partial y^{2}}+v\frac{\partial^{3}u}{\partial y^{3}}-\frac{\partial u}{\partial y}\frac{\partial^{2}u}{\partial x\partial y}+\frac{\partial u}{\partial x}\frac{\partial^{2}u}{\partial y^{2}}\right)-\frac{\nu }{K^{*}}u-Fu^{2},\;$$ (4)

$$\rho c_{p}\left(u\frac{\partial T}{\partial x}+v\frac{\partial T}{\partial y}\right)=-\nabla .q,\;$$ (5)

$$u\frac{\partial a}{\partial x}+v\frac{\partial a}{\partial y}=D_{A}\frac{\partial^{2}a}{\partial y^{2}}-k_{c}ab^{2},\;$$ (6)

$$u\frac{\partial b}{\partial x}+v\frac{\partial b}{\partial y}=D_{B}\frac{\partial^{2}b}{\partial y^{2}}+k_{c}ab^{2}.$$ (7)

Note that *u* and *v* denote the fluid velocities in *x* − and *y* −directions respectively while *v* (= *μ* / *ρ*), *μ* and *ρ* stand for kinematic viscosity, dynamic viscosity and density of base liquid respectively, *k*_0_ = −*α*_1_ / *ρ* for elastic parameter, *T* for temperature, K* for permeability of porous media, $F=C_{b}/xK^{{*}^{1/2}}$ for non-uniform inertia coefficient of porous medium, *C*_*b*_ for drag coefficient and **q** for heat flux. Here *k*_0_ > 0 represents elastico-viscous fluid, *k*_0_ < 0 is for second grade fluid and *k*_0_ = 0 for Newtonian fluid. According to Cattaneo-Christov heat flux theory, we have [21, 27]:

$$q+\lambda \left(\frac{\partial q}{\partial t}+V.\nabla q-q.\nabla V+\left(\nabla .V\right)\;q\right)=-k\nabla T,\;$$ (8)

where *k* stands for thermal conductivity and *λ* for relaxation time of heat flux. Classical Fourier's law is deduced by putting *λ* = 0 in Eq (8). By considering the incompressibility condition (∇.**V** = 0) and steady flow with $(\frac{\partial q}{\partial t}=0),$ Eq (8) becomes [21, 27]:

q+λ(V.∇q−q.∇V)=−k∇T. (9)

Then the energy equation takes the following form [21, 27]:

$$u\frac{\partial T}{\partial x}+v\frac{\partial T}{\partial y}+\lambda \Phi_{E}=\alpha \left(\frac{\partial^{2}T}{\partial y^{2}}\right),\;$$ (10)

$$\Phi_{E}=u\frac{\partial u}{\partial x}\frac{\partial T}{\partial x}+v\frac{\partial v}{\partial y}\frac{\partial T}{\partial y}+u\frac{\partial v}{\partial x}\frac{\partial T}{\partial y}+v\frac{\partial u}{\partial y}\frac{\partial T}{\partial x}+2uv\frac{\partial^{2}T}{\partial x\partial y}+u^{2}\frac{\partial^{2}T}{\partial x^{2}}+v^{2}\frac{\partial^{2}T}{\partial y^{2}}.$$ (11)

The associated boundary conditions are [27, 38]:

$$u=u_{w}\left(x\right)=cx,\;v=0,\;T=T_{w},\;D_{A}\frac{\partial a}{\partial y}=k_{s}a,\;D_{B}\frac{\partial b}{\partial y}=-k_{s}a\text{ at }y=0,\;$$ (12)

$$u\to 0,\;T\to T_{\infty },\;a\to a_{0},\;b\to 0\text{ as }y\to \infty ,\;$$ (13)

in which *α* = *k* /(*ρc*_*p*_) stands for thermal diffusivity, *D*_*A*_ and *D*_*B*_ for diffusion coefficients, *T*_*w*_ for constant surface temperature, *T*_∞_ for ambient fluid temperature and *c* for positive stretching rate constant with *T*^−1^ as the dimension. Selecting

$$\begin{array}{c} u=cxf^{\prime }(\zeta ),\;v=-{\left(c\nu \right)}^{1/2}f(\zeta ),\;\zeta ={\left(\frac{c}{\nu }\right)}^{1/2}y,\\ \theta (\zeta )=\frac{T-T_{\infty }}{T_{w}-T_{\infty }},\;a=a_{0}\phi (\zeta ),\;b=a_{0}h(\zeta ). \end{array}$$ (14)

Continuity equation is trivially satisfied and Eqs (4)–(13) become

$$f^{\prime \prime \prime }+ff^{\prime \prime }-k_{1}^{*}\left(2f^{\prime }f^{\prime \prime \prime }-f^{\prime \prime^{2}}-ff^{iv}\right)-\lambda f^{\prime }-(1+F_{r})f^{\prime^{2}}=0,\;$$ (15)

$$\frac{1}{\text{Pr}}\theta^{\prime \prime }+f\theta^{\prime }-\gamma \left(ff^{\prime }\theta^{\prime }+f^{2}\theta^{\prime \prime }\right)=0,\;$$ (16)

$$\frac{1}{Sc}\phi^{\prime \prime }+f\phi^{\prime }-K\phi h^{2}=0,\;$$ (17)

$$\frac{\delta }{Sc}h^{\prime \prime }+fh^{\prime }+K\phi h^{2}=0,\;$$ (18)

$$f=0,\;f^{\prime }=1,\;\theta =1,\;\phi \prime =K_{s}\phi ,\;\delta h^{\prime }=-K_{s}\phi \text{ at }\zeta =0,\;$$ (19)

$$f^{\prime }\to 0,\;\theta \to 0,\;\phi \to 1,\;h\to 0\text{ as }\zeta \to \infty ,\;$$ (20)

where $k_{1}^{*}$ stands for viscoelastic parameter, *λ* for porosity parameter, *F*_*r*_ for inertia coefficient, Pr for Prandtl number, *γ* for thermal relaxation parameter, *Sc* for Schmidt number, *K* for strength of homogeneous reaction, *δ* for ratio of diffusion coefficients and *K*_*s*_ for strength of heterogeneous reaction. These parameters can be specified by using the definitions given below:

$$\begin{array}{c} k_{1}^{*}=-\frac{k_{0}c}{\nu },\text{ Pr}=\frac{\nu }{\alpha },\;\gamma =c\lambda ,\;\\ F_{r}=\frac{C_{b}}{K^{{*}^{1/2}}},\;Sc=\frac{\nu }{D_{A}},\;K=\frac{k_{c}a_{0}^{2}}{u_{w}},\;K_{s}=\frac{k_{s}}{D_{A}a_{0}}\sqrt{\frac{c}{\nu }},\;\delta =\frac{D_{B}}{D_{A}}. \end{array}\}\;$$ (21)

When *D*_*A*_ = *D*_*B*_ then *δ* = 1 and thus

ϕ(ζ)+h(ζ)=1. (22)

Now Eqs (17) and (18) yield

$$\frac{1}{Sc}\phi^{\prime \prime }+f\phi^{\prime }-K\phi {\left(1-\phi \right)}^{2}=0.$$ (23)

The subjected boundary conditions are

$$\phi^{\prime }\left(0\right)=K_{s}\phi \left(0\right),\;\phi \left(\infty \right)\to 1.$$ (24)

Skin friction coefficient is defined as follows [27]:

$$C_{f}=\frac{{\tau_{w}|}_{y=0}}{\rho u_{w}^{2}}=\frac{{\left(\nu \frac{\partial u}{\partial y}-k_{0}\left(u\frac{\partial^{2}u}{\partial x\partial y}-2\frac{\partial u}{\partial y}\frac{\partial v}{\partial y}+v\frac{\partial^{2}u}{\partial y^{2}}\right)\right)}_{y=0}}{u_{w}^{2}}.$$ (25)

Skin friction coefficient through dimensionless scale is

$$Re_{x}^{1/2}C_{f}=\left(1-3k_{1}^{*}\right)\;f^{\prime \prime }\left(0\right).$$ (26)

Local Nusselt number is given by

$$Re_{x}^{-1/2}Nu_{x}=-\theta^{\prime }\left(0\right),\;$$ (27)

in which Re_*x*_ = *u*_*w*_*x*/*v* depicts the local Reynolds number.

## 3. Solutions by OHAM

The series solutions of Eqs (15), (16) and (23) through the boundary conditions (19), (20) and (24) have been developed by employing optimal homotopy analysis technique (OHAM). Initial approximations and linear operators are

$$f_{0}(\zeta )=1-e^{-\zeta },\;\theta_{0}(\zeta )=e^{-\zeta },\;\phi_{0}(\eta )=1-\frac{1}{2}e^{-K_{s}\zeta },\;$$ (28)

$$L_{f}=\frac{d^{3}f}{d\zeta^{3}}-\frac{df}{d\zeta },\;L_{\theta }=\frac{d^{2}\theta }{d\zeta^{2}}-\theta ,\;L_{\phi }=\frac{d^{2}\phi }{d\zeta^{2}}-\phi .$$ (29)

The above linear operators have the characteristics given as under

$$L_{f}\left[C_{1}^{**}+C_{2}^{**}e^{\zeta }+C_{3}^{**}e^{-\zeta }\right]=0,\;L_{\theta }\left[C_{4}^{**}e^{\zeta }+C_{5}^{**}e^{-\zeta }\right]=0,\;L_{\phi }\left[C_{6}^{**}e^{\zeta }+C_{7}^{**}e^{-\zeta }\right]=0,\;$$ (30)

in which $C_{j}^{**}$ (*j* = 1 − 7) stands for arbitrary constants.

## 4. Optimal convergence control parameters

Here the nonzero auxiliary parameters ℏ_*f*_, ℏ_*θ*_ and ℏ_*ϕ*_ in homotopic solutions regulate the convergence region and also rate of homotopy solutions. To obtain the optimal values of ℏ_*f*_, ℏ_*θ*_ and ℏ_*ϕ*_, we have employed the idea of minimization by defining the average squared residual errors as proposed by Liao [49].

$$\varepsilon_{m}^{f}=\frac{1}{k+1}\overset{k}{\underset{j=0}{\sum }}{\left[N_{f}{\left(\overset{m}{\underset{i=0}{\sum }}\hat{f}\left(\zeta \right)\right)}_{\zeta =j\delta \zeta }\right]}^{2},\;$$ (31)

$$\varepsilon_{m}^{\theta }=\frac{1}{k+1}\overset{k}{\underset{j=0}{\sum }}{\left[N_{\theta }{\left(\overset{m}{\underset{i=0}{\sum }}\hat{f}\left(\zeta \right),\;\overset{m}{\underset{i=0}{\sum }}\hat{\theta }\left(\zeta \right)\right)}_{\zeta =j\delta \zeta }\right]}^{2},\;$$ (32)

$$\varepsilon_{m}^{\phi }=\frac{1}{k+1}\overset{k}{\underset{j=0}{\sum }}{\left[N_{\phi }{\left(\overset{m}{\underset{i=0}{\sum }}\hat{f}\left(\zeta \right),\;\overset{m}{\underset{i=0}{\sum }}\hat{\phi }\left(\zeta \right)\right)}_{\zeta =j\delta \zeta }\right]}^{2}.$$ (33)

**Fig 1:**
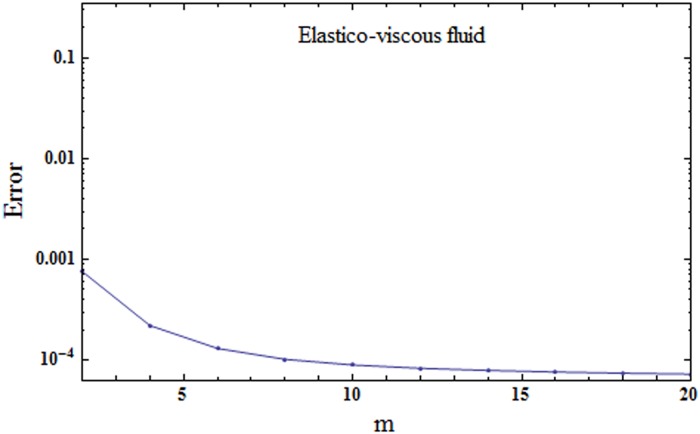
Total residual error for elastico-viscous fluid.

**Fig 2:**
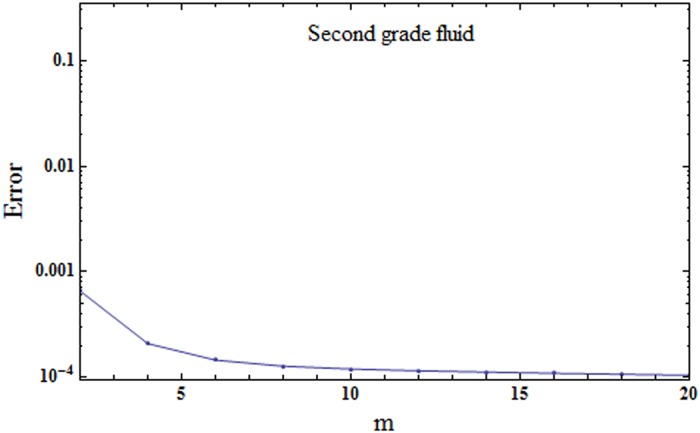
Total residual error for second grade fluid.

Following Liao [49]:

$$\varepsilon_{m}^{t}=\varepsilon_{m}^{f}+\varepsilon_{m}^{\theta }+\varepsilon_{m}^{\phi },\;$$ (34)

where $\varepsilon_{m}^{t}$ stands for total squared residual error, *δζ* = 0.5 and *k* = 20. The optimal values of convergence control parameters at 2nd order of approximations for elastico-viscous fluid case are *h*_*f*_ = − 1.53374, *h*_*θ*_ = − 1.30423 and *h*_*ϕ*_ = − 1.7072 and total averaged squared residual error is $\varepsilon_{m}^{t}=7.6\times 10^{-4}$ while the optimal values of convergence control parameters at 2nd order of approximations for second grade fluid case are *h*_*f*_ = − 1.04537, *h*_*θ*_ = − 1.29762 and *h*_*ϕ*_ = − 1.75305 and total averaged squared residual error is $\varepsilon_{m}^{t}=6.6\times 10^{-4}$. Figs 1 and 2 show the corresponding total residual error graphs. Tables 1 and 2 present the individual average squared residual errors using optimal values of convergence control parameters at *m* = 2. It is clearly observed that the averaged squared residual errors reduce with higher order approximations.

**Table 1:** Individual averaged squared residual errors for elastico-viscous fluid considering optimal values of auxiliary parameters.

*m*	$\varepsilon_{m}^{f}$	$\varepsilon_{m}^{\theta }$	$\varepsilon_{m}^{\phi }$
2	3.75 × 10^−5^	6.24 × 10^−4^	9.80 × 10^−5^
6	3.76 × 10^−6^	4.15 × 10^−5^	8.51 × 10^−5^
10	1.33 × 10^−6^	8.41 × 10^−6^	7.95 × 10^−5^
16	5.01 × 10^−7^	1.49 × 10^−6^	7.40 × 10^−5^
20	3.22 × 10^−7^	6.10 × 10^−7^	7.14 × 10^−5^

**Table 2:** Individual averaged squared residual errors for second grade fluid considering optimal values of auxiliary parameters.

*m*	$\varepsilon_{m}^{f}$	$\varepsilon_{m}^{\theta }$	$\varepsilon_{m}^{\phi }$
2	8.83 × 10^−7^	5.23 × 10^−4^	1.32 × 10^−4^
6	1.27 × 10^−8^	2.46 × 10^−5^	1.21 × 10^−4^
10	6.63 × 10^−9^	5.98 × 10^−6^	1.14 × 10^−4^
16	4.39 × 10^−9^	3.15 × 10^−6^	1.06 × 10^−4^
20	3.42 × 10^−9^	2.57 × 10^−6^	1.02 × 10^−4^

## 5. Discussion

This section has been arranged to explore the effects of various influential parameters on the velocity *f'*(*ζ*), temperature *θ*(*ζ*) and concentration *ϕ*(*ζ*) distributions. Here the elastico-viscous $\left(k_{1}^{*}>0\right)$ and second grade $\left(k_{1}^{*}<0\right)$ fluids are considered. The computations have been carried out for various values of $k_{1}^{*}\left(-0.2\leq k_{1}^{*}\leq 0.3\right),$ *λ*(0.0 ≤ *λ* ≤ 0.7), *F*_*r*_ (0.0 ≤ *F*_*r*_ ≤ 0.9), *γ* (0.0 ≤ *γ* ≤ 0.5), Pr(0.7 ≤ Pr ≤ 1.3), *Sc*(0.5 ≤ *Sc* ≤ 1.5), *K*(0.5 ≤ *K* ≤ 1.3) and *K*_*s*_(0.13 ≤ *K*_*s*_ ≤ 0.5) [59].

**Fig 3:**
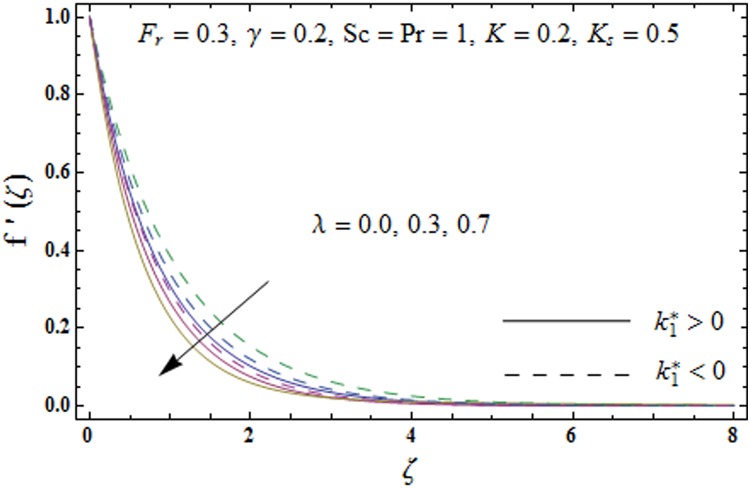
Plots of velocity field *f'*(*ζ*) for porosity parameter *λ*.

**Fig 4:**
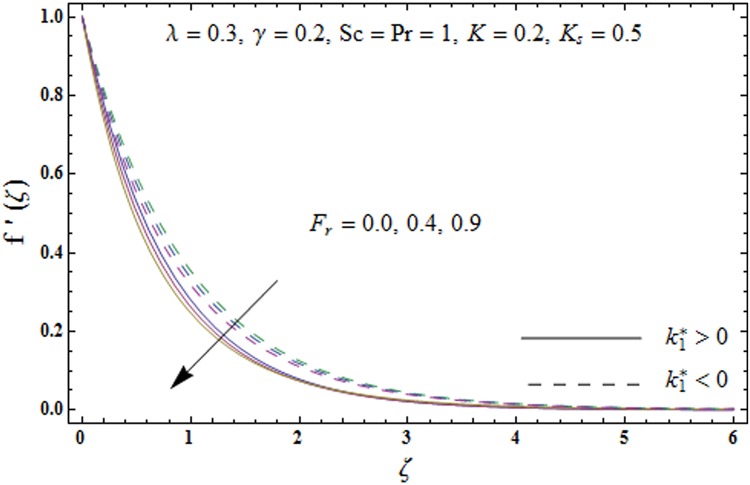
Plots of velocity field *f'*(*ζ*) for inertia coefficient *F*_*r*_.

Impacts of porosity parameter *λ* and inertia coefficient *F*_*r*_ on the non-dimensional velocity field *f'*(*ζ*) are plotted in Figs 3 and 4 respectively. Fig 3 depicts the impact of porosity parameter *λ* on velocity field *f'*(*ζ*) for both elastico-viscous and second grade fluids. An increment in the values of porosity parameter *λ* present decreasing trend in the velocity field *f'*(*ζ*) for both fluids. Physically the existence of porous media is to increase the resistance to fluid flow which causes decay in fluid velocity and related momentum layer thickness. Fig 4 presents change in velocity field *f'*(*ζ*) for varying inertia coefficient *F*_*r*_ for both elastico-viscous and second grade fluids. It has been noticed that the velocity profile *f'*(*ζ*) reduces for higher values of inertia coefficient *F*_*r*_.

**Fig 5:**
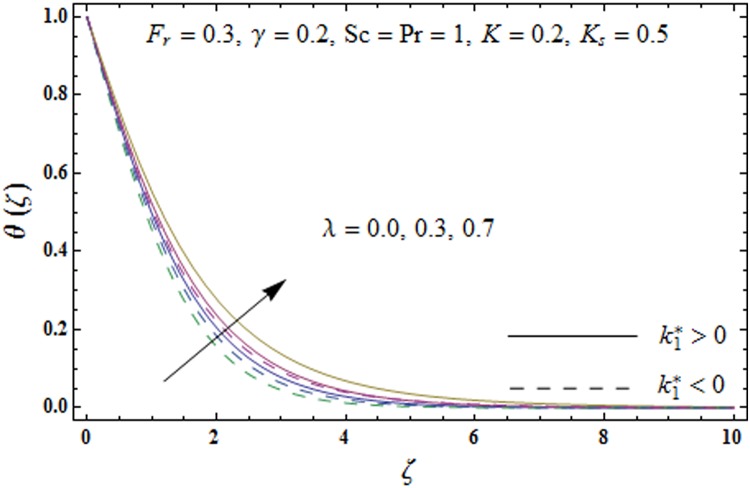
Plots of temperature field *θ*(*ζ*) for porosity parameter *λ*.

**Fig 6:**
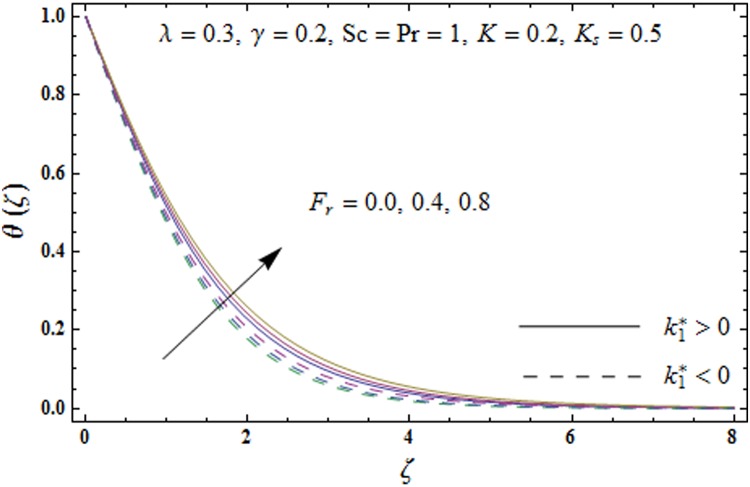
Plots of temperature field *θ*(*ζ*) for inertia coefficient *F*_*r*_.

**Fig 7:**
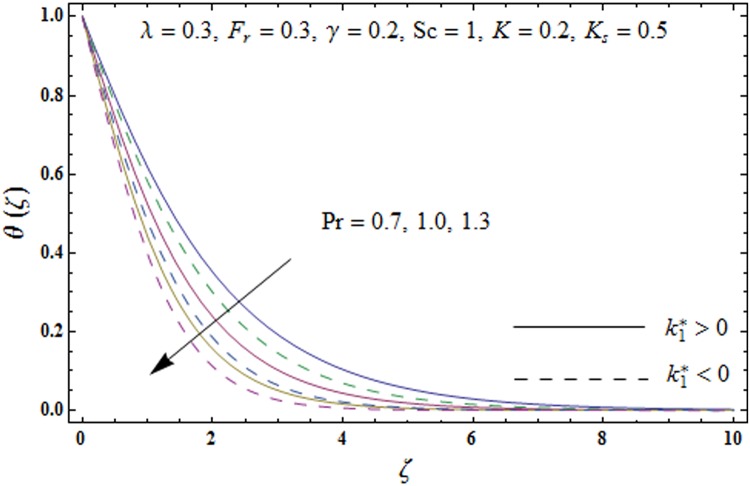
Plots of temperature field *θ*(*ζ*) for Prandtl number Pr.

**Fig 8:**
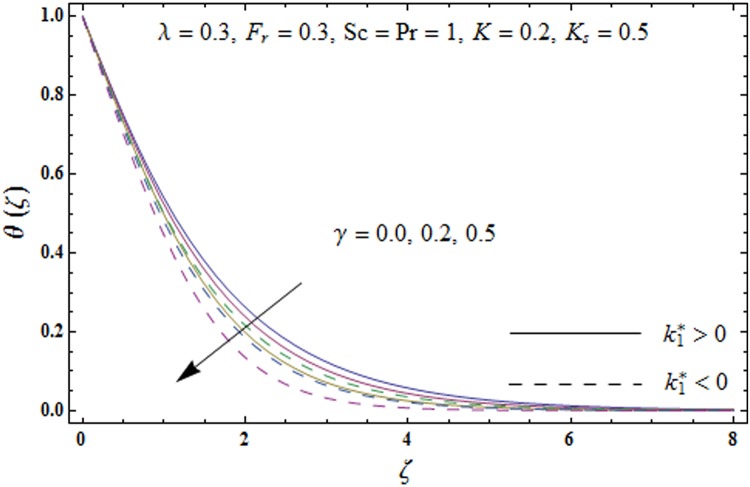
Plots of temperature field *θ*(*ζ*) for thermal relaxation parameter *γ*.

Figs 5–8 are plotted to explore the impacts of porosity parameter *λ*, inertia coefficient *F*_*r*_, thermal relaxation parameter *γ* and Prandtl number Pr on the non-dimensional temperature field *θ*(*ζ*). Fig 5 displays the change in temperature field *θ*(*ζ*). for varying porosity parameter *λ*. Higher values of porosity parameter *λ* correspond to stronger temperature field *θ*(*ζ*) and more thermal layer thickness for both fluids. Fig 6 presents the effect of inertia coefficient *F*_*r*_ on temperature field *θ*(*ζ*) It has been noticed that the temperature field *θ*(*ζ*) and related thermal layer thickness are more for increasing values of inertia coefficient *F*_*r*_ for both fluids. Fig 7 presents that how Prandtl number Pr effect the temperature field *θ*(*ζ*). It has been observed that the temperature field *θ*(*ζ*) and thermal layer thickness are lower for increasing values of Prandtl number Pr for both elastico-viscous and second grade fluids Effect of thermal relaxation parameter *γ* on temperature field *θ*(*ζ*) is sketched in Fig 8 for both fluids. Both temperature field *θ*(*ζ*) and thermal layer thickness are reduced when thermal relaxation parameter *γ* increases.

**Fig 9:**
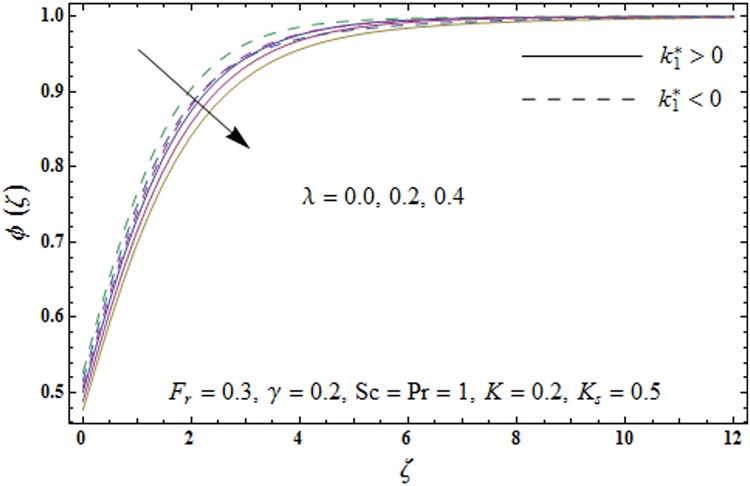
Plots of concentration field *ϕ*(*ζ*) for porosity parameter *λ*.

**Fig 10:**
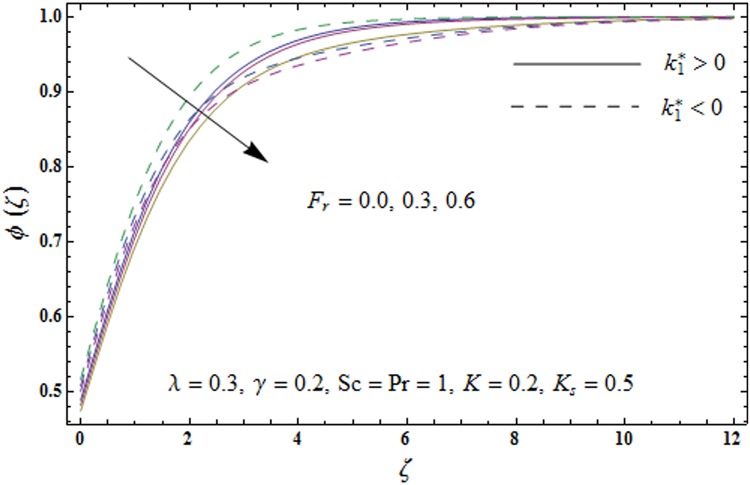
Plots of concentration field *ϕ*(*ζ*) for inertia coefficient *F*_*r*_.

**Fig 11:**
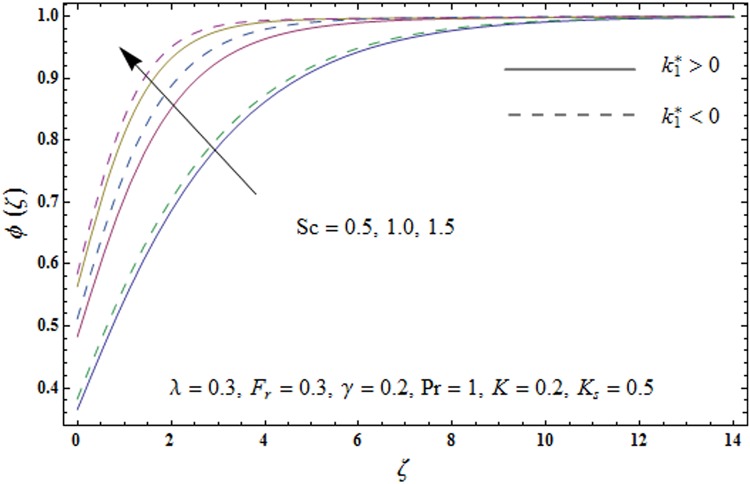
Plots of concentration field *ϕ*(*ζ*) for Schmidt number *Sc*.

**Fig 12:**
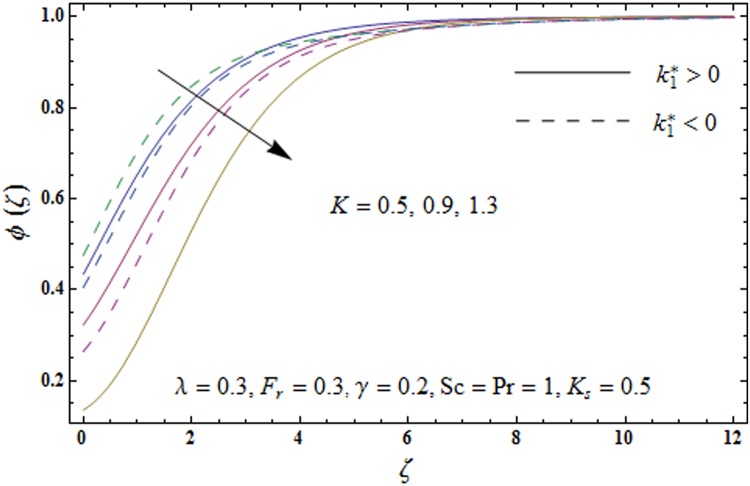
Plots of concentration field *ϕ*(*ζ*) for strength of homogeneous reaction *K*_*s*_.

**Fig 13:**
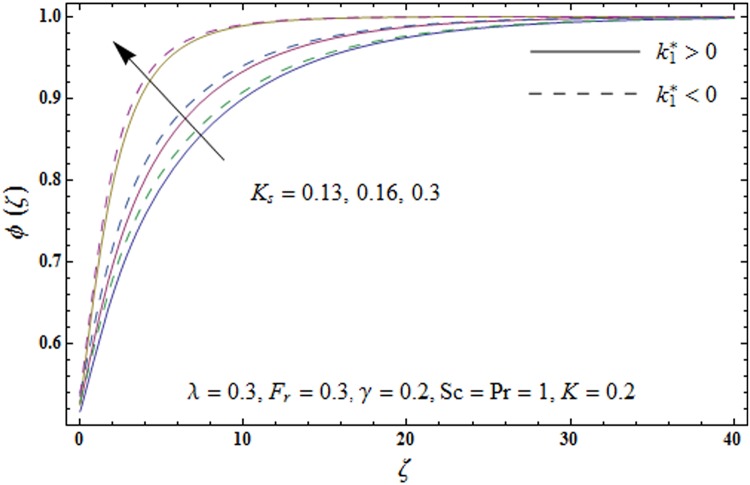
Plots of concentration field *ϕ*(*ζ*) for strength of heterogeneous reaction *K*_*s*_.

Figs 9–13 are sketched to analyze the impacts of porosity parameter *λ*, inertia coefficient *F*_*r*_, Schmidt number *Sc*, strength of homogeneous reaction *K* and strength of heterogeneous reaction *K*_*s*_ on the non-dimensional concentration field *ϕ*(*ζ*). Fig 9 plots the concentration field *ϕ*(*ζ*) for varying porosity parameter *λ*. It has been noticed that by increasing porosity parameter *λ*, a reduction in concentration field *ϕ*(*ζ*) is observed for both elastico-viscous and second grade fluids Fig 10 depicts the effect of inertia coefficient *F*_*r*_ on concentration field *ϕ*(*ζ*). Higher values of inertia coefficient *F*_*r*_ lead to weaker concentration field *ϕ*(*ζ*) and less concentration layer thickness for both elastico-viscous and second grade fluids. Fig 11 demonstrates that how Schmidt number *Sc* effect the concentration field *ϕ*(*ζ*). Concentration field *ϕ*(*ζ*) shows increasing trend for larger Schmidt number *Sc* for both elastico-viscous and second grade fluids. Fig 12 shows that how strength of homogeneous reaction *K* effect the concentration field *ϕ*(*ζ*) for both elastico-viscous and second grade fluids. It has been observed that larger *K* produces decreasing trend in concentration field *ϕ*(*ζ*). Fig 13 depicts the impact of *K*_*s*_ on concentration field *ϕ*(*ζ*) for both elastico-viscous and second grade fluids Larger *K*_*s*_ shows increasing trend for concentration field *ϕ*(*ζ*).

Table 3 is arranged to analyze the skin friction coefficient $Re_{x}^{1/2}C_{f}$ for varying $k_{1}^{*},$ *λ* and *F*_*r*_. It is noticed that the skin friction coefficient has higher values for larger porosity parameter *λ* and inertia coefficient *F*_*r*_ in the presence of both elastico-viscous and second grade fluids. Table 4 shows the comparison for different values of viscoelastic parameter $k_{1}^{*}$ with homotopy analysis method (HAM). Table 4 presents a good agreement of OHAM solution with the existing homotopy analysis method (HAM) solution in a limiting case. Tables 5 and 6 present the numerical data for local Nusselt number −*θ'*(0) for distinct values of *γ* for both elastico-viscous $\left(k_{1}^{*}>0\right)$ and second grade $\left(k_{1}^{*}<0\right)$ fluids respectively. Local Nusselt number is noted high via *γ* in the presence of both elastico-viscous and second grade fluids.

**Table 3:** Numerical data for skin friction coefficient $-Re_{x}^{1/2}C_{f}$ for different values of $k_{1}^{*},$ *λ* and *F*_*r*_.

		$-Re_{x}^{1/2}C_{f}$	$-Re_{x}^{1/2}C_{f}$
λ	*F*_*r*_	$k_{1}^{*}\;=\;-0.2$	$k_{1}^{*}\;=\;-0.2$
**0.0**	**0.3**	1.5837	0.4934
0.1		1.6503	0.5134
0.2		1.7142	0.5327
0.3	0.0	1.6653	0.5099
	0.1	1.7028	0.5240
	0.2	1.7396	0.5378

**Table 4:** Comparative values of $-Re_{x}^{1/2}C_{f}$ for different values of viscoelastic parameter $k_{1}^{*}$ when *λ = F*_*r*_ = 0.

$k_{1}^{*}$	$-Re_{x}^{1/2}C_{f}$	
	*OHAM*	*HAM*[27]
0.0	1.00000	1.00000
0.1	0.73786	0.73786
0.2	0.44721	0.44721
0.3	0.11952	0.11952

**Table 5:** Numerical data of local Nusselt number − *θ'*(0) in elastico-viscous fluid for different values of *γ* when $k_{1}^{*}=0.2,$ *λ* = *F*_*r*_ = 0.3, Pr = *Sc* = 1.0, *K* = 0.2 and *K*_*s*_ = 0.5.

*γ*	0.0	0.2	0.4	0.6	0.8
− *θ'*(0)	0.5167	0.5299	0.5437	0.5586	0.5747

**Table 6:** Numerical data of local Nusselt number − *θ'*(0) in second grade fluid for different values of *γ* when $k_{1}^{*}=-0.2,$ *λ* = *F*_*r*_ = 0.3, Pr = *Sc* = 1.0, *K* = 0.2 and *K*_*s*_ = 0.5.

*γ*	0.0	0.2	0.4	0.6	0.8
− *θ'*(0)	0.5653	0.5822	0.6001	0.6193	0.6411

## 6. Conclusions

Darcy-Forchheimer flow of viscoelastic fluids bounded by a linear stretchable surface with Cattaneo-Christov heat flux model and homogeneous-heterogeneous reactions has been discussed. The key findings of present investigation are listed below:

- Increasing values of porosity parameter *λ* and inertia coefficient *F*_*r*_ show reduction in the velocity field *f'*(*ζ*).
- Both temperature *θ*(*ζ*) and concentration *ϕ*(*ζ*) fields depict opposite behavior for larger porosity parameter *λ*.
- Larger values of inertia coefficient *F*_*r*_ lead to higher temperature field *θ*(*ζ*) while opposite trend is noticed for concentration field *ϕ*(*ζ*).
- Larger Prandtl number Pr and thermal relaxation parameter *γ* show decay in temperature *θ*(*ζ*).
- An increment in Schmidt number *Sc* and strength of heterogenous reaction *K*_*s*_ leads to higher concentration field *ϕ*(*ζ*).
- Larger strength of homogeneous reaction *K* causes a decay in the concentration field *ϕ*(*ζ*).
- Skin friction coefficient for both fluids is higher for larger porosity parameter *λ* and inertia coefficient *F*_*r*_.
- Local Nusselt number is increasing function of thermal relaxation parameter *γ* in cases of both fluids.

The present analysis provides a motivation for future developments on the topic in regimes of shrinking sheet, variable sheet thickness, variable thermal conductivity and Cattaneo-Christov double diffusion [60].
